# Anti-CD45 Radioimmunotherapy with ^90^Y but Not ^177^Lu Is Effective Treatment in a Syngeneic Murine Leukemia Model

**DOI:** 10.1371/journal.pone.0113601

**Published:** 2014-12-02

**Authors:** Johnnie J. Orozco, Ethan R. Balkin, Ted A. Gooley, Aimee Kenoyer, Donald K. Hamlin, D. Scott Wilbur, Darrell R. Fisher, Mark D. Hylarides, Mazyar Shadman, Damian J. Green, Ajay K. Gopal, Oliver W. Press, John M. Pagel

**Affiliations:** 1 Clinical Research Division, Fred Hutchinson Cancer Research Center, Seattle, WA, United States of America; 2 Hematology Division, University of Washington, Seattle, WA, United States of America; 3 Radiation Oncology, University of Washington, Seattle, WA, United States of America; 4 Dade Moeller Health Group, Richland, WA, United States of America; 5 Medical Oncology, University of Washington, Seattle, WA, United States of America; Queen's University Belfast, United Kingdom

## Abstract

Radioimmunotherapy (RIT) for treatment of hematologic malignancies has primarily employed monoclonal antibodies (Ab) labeled with ^131^I or ^90^Y which have limitations, and alternative radionuclides are needed to facilitate wider adoption of RIT. We therefore compared the relative therapeutic efficacy and toxicity of anti-CD45 RIT employing ^90^Y and ^177^Lu in a syngeneic, disseminated murine myeloid leukemia (B6SJLF1/J) model. Biodistribution studies showed that both ^90^Y- and ^177^Lu-anti-murine CD45 Ab conjugates (DOTA-30F11) targeted hematologic tissues, as at 24 hours 48.8±21.2 and 156±14.6% injected dose per gram of tissue (% ID/g) of ^90^Y-DOTA-30F11 and 54.2±9.5 and 199±11.7% ID/g of ^177^Lu-DOTA-30F11 accumulated in bone marrow (BM) and spleen, respectively. However, ^90^Y-DOTA-30F11 RIT demonstrated a dose-dependent survival benefit: 60% of mice treated with 300 µCi ^90^Y-DOTA-30F11 lived over 180 days after therapy, and mice treated with 100 µCi ^90^Y-DOTA-30F11 had a median survival 66 days. ^90^Y-anti-CD45 RIT was associated with transient, mild myelotoxicity without hepatic or renal toxicity. Conversely, ^177^Lu- anti-CD45 RIT yielded no long-term survivors. Thus, ^90^Y was more effective than ^177^Lu for anti-CD45 RIT of AML in this murine leukemia model.

## Introduction

Acute myeloid leukemia (AML) is associated with high rates of relapse and mortality and despite aggressive treatments such as hematopoietic cell transplantation (HCT) many patients fail to achieve long-term survival. Attempts to decrease relapse after HCT have, among other approaches, utilized intensified cytoreductive therapies either by increasing total body irradiation (TBI) or doses of chemotherapy during HCT conditioning. Escalated TBI doses for HCT preparative regimens have led to fewer relapses, but these efforts have typically not translated into improved overall survival (OS) because of increased treatment-related mortality [1]–[3]. In contrast, the use of radiolabeled monoclonal antibodies (Ab) directed at cell surface antigens allow for the targeted delivery of escalated doses of radiation to bone marrow (BM), spleen, and other sites of malignancy while sparing normal organs [4]–[11]. In addition, RIT may improve outcomes when used in combination with chemotherapy and/or HCT [10], [12]–[14]. Though leukemia cells express multiple surface antigens that could be targeted, clinical RIT trials to treat AML have primarily used anti-CD33, anti-CD66 and anti-CD45 Ab as vehicles to deliver radiotherapy. CD45 is present on more than 70% of nucleated cells in normal BM, and on more than 85% of leukemic samples [15]–[17], with an average copy number of ∼200,000 molecules per cell [18].

The radionuclides employed in RIT to date have limitations. We have used iodine-131 (^131^I) in our clinical and pre-clinical studies because there is extensive experience with its medical use, the technology for radiolabeling Abs with iodine is well established, and its gamma component allows direct determination of labeled Ab biodistribution. However, the high-energy gamma component of ^131^I requires that patients be treated in radiation isolation, and poses a radiation exposure risk for staff and family. In addition, not all facilitates are capable of handling and disposing of ^131^I waste. To supplant ^131^I-anti-CD45 Ab an alternative radionuclide yttrium-90 (^90^Y) has been selected as a therapeutic radioisotope for our studies because it is a pure β-emitter that is commercially available in high specific activity and purity. Moreover, ^90^Y has a high-energy tissue penetration. However, ^90^Y cannot be imaged directly for which an imaging surrogate for dosimetry studies is required for ^90^Y. Therefore, a need remains for alternative radionuclides that can be used for imaging procedures, with adequate energy profiles to achieve therapeutic effects. Lutetium-177 (^177^Lu) potentially fulfills this need as its beta-emission energy, path length, and half-life are similar to the efficacious ^131^I. However, unlike ^131^I, ^177^Lu has lower and safer energy gamma-emissions that do not require isolation, and facilitate imaging for dosimetry. In addition, ^177^Lu with a shorter path length (0.9 mm) offers the potential for less non-specific toxicity compared to ^90^Y (path length  = 2.7 mm). We hypothesized that ^177^Lu may be an efficacious alternative radionuclide to ^90^Y for the treatment of hematologic malignancies with anti-CD45 RIT. In these studies we compared the therapeutic efficacy and toxicity of ^177^Lu- and ^90^Y-anti-CD45 RIT as primary treatment in an immunocompetent, syngeneic murine myeloid leukemia model, and showed that ^90^Y was more effective than ^177^Lu for anti-CD45 RIT of AML.

## Methods

### Mice

Female B6SJLF1/J mice (6 to 12 weeks old) were purchased from Jackson Laboratories (Bar Harbor, ME). Imaging studies used female athymic mice (6 to 12 weeks old) from Harlan Laboratories (Indianapolis, IN). Mice were housed at the FHCRC animal care facility in a pathogen-free environment, and handled by protocols approved by the FHCRC Institutional Animal Care and Use Committee (IACUC IR #1716). This study was carried out in strict accordance with the recommendations in the Guide for the Care and Use of Laboratory Animals of the National Institutes of Health, and all efforts were made to minimize suffering.

### Cell Lines, Antibodies and Radiolabeling

Murine AML cells were produced as previously described by serial passage in SJL/J mice [19], [20]. Control Ab (polyclonal rat IgG) was purchased from Sigma Aldrich (St. Louis, MO). Rat IgG2b anti-murine CD45 Ab (30F11) was purified from mouse ascites, and DOTA-30F11 and DOTA-rat IgG were prepared as previously described [21]. Yttrium-90 was purchased from PerkinElmer, Inc. (Waltham, MA), while ^177^Lu was acquired from either PerkinElmer, Inc. or the University of Missouri Research Reactor (MURR, Columbia, MO). DOTA-30F11 and DOTA-rat IgG were radiolabeled with ^177^Lu or ^90^Y as previously described [22], and were PD10 column (Bio-Rad, Hercules, CA) purified, resulting in labeling efficiencies for all ^90^Y- and ^177^Lu-DOTA-Ab injectates of >90%. Radiochemical purity determined by HPLC was >99% for each radiolabeled DOTA-30F11 construct.

### Biodistribution Studies

The percent injected dose of radioactivity per gram of tissue (% ID/g) for ^90^Y- and ^177^Lu-DOTA-30F11, with correction for radioactive decay, were performed in groups of five mice, as previously described [19].

### Cerenkov Imaging

Imaging studies used female athymic mice from Harlan Laboratories (Indianapolis, IN). Radiolabeled DOTA-Ab (0.67 nmol) (100 µCi of either radionuclide) was delivered *via* tail vein to up to five athymic nude mice at time  = 0 hours. Mice were anesthetized and imaged with 2.5% isoflurane, using a Xenogen IVIS Spectrum platform from PerkinElmer, Inc. (Waltham, MA). Images were acquired with Living Image software (v 3.0, PerkinElmer, Inc.) at 0.25, 1, 2, 4, 24, and 48 hours after delivery of each radiolabeled Ab with camera settings of: medium binning, f-stop 1, open filter channel, and image acquisition length of 15 seconds (^90^Y) or 2 minutes (^177^Lu). The fluorescence intensity scale was determined using background-corrected fluorescent whole-body images measured automatically by the software.

### Radioimmunotherapy of Leukemic Mice

RIT studies were performed in groups of 10 mice as previously described with 100 or 300 µCi ^90^Y- or ^177^Lu-DOTA-30F11, or with 300 µCi of either ^90^Y- or ^177^Lu-DOTA-rat IgG [19], [20]. Briefly, mice were placed on a Uniprim-containing diet (irradiated, 4100 ppm; Animal Specialties, Hubbard, OR) 3 days before *i.v.* injection with 10^5^ SJL leukemic cells. Two days later mice received radiolabeled DOTA-30F11 or DOTA-rat IgG. Mice were monitored daily after RIT injections, and weighed 2–3 times per week. Mice were sacrificed in a CO_2_ chamber per IACUC protocols for >30% weight loss, for severe lethargy or if significantly moribund.

### Radiation Dosimetry

Using biodistribution data and the standard Medical Internal Radiation Dose Methods [23], radiation absorbed doses for blood and each organ were calculated as described previously [24], [25].

### Toxicity Assessments

Groups of 10 non-leukemia bearing B6SJLF1/J mice were given 300 µCi ^90^Y-DOTA-30F11, or 300 or 665 µCi ^177^Lu-DOTA-30F11 by tail vein injection. Mice were bled *via* the retro-orbital plexus at baseline, 1, 2, 3, 4, and 8 weeks after injection of radiolabeled Ab. Five mice per group had blood analyzed for complete blood counts, and the other 5 mice had blood analyzed for kidney and liver function. Values were followed serially and compared to those from untreated age-matched control B6SJLF1/J mice.

### Biostatistics

Comparisons were made among groups of 5 mice or 10 mice. Five mice per group provide 80% power to observe a statistically significant difference (at the 2-sided significance level of.05) in a continuous outcome if the true difference between groups is 2.02 standard-deviation units; 10 mice per group provide 80% power if the true difference is 1.33 standard-deviation units. The two-sample t-test was used to compare continuous outcomes, and the log-rank test was used to compare survival between groups. In addition, 95% confidence intervals for the difference between groups were given when comparing the difference between groups.

## Results

### Comparative Biodistribution Studies

**Figure 1:**
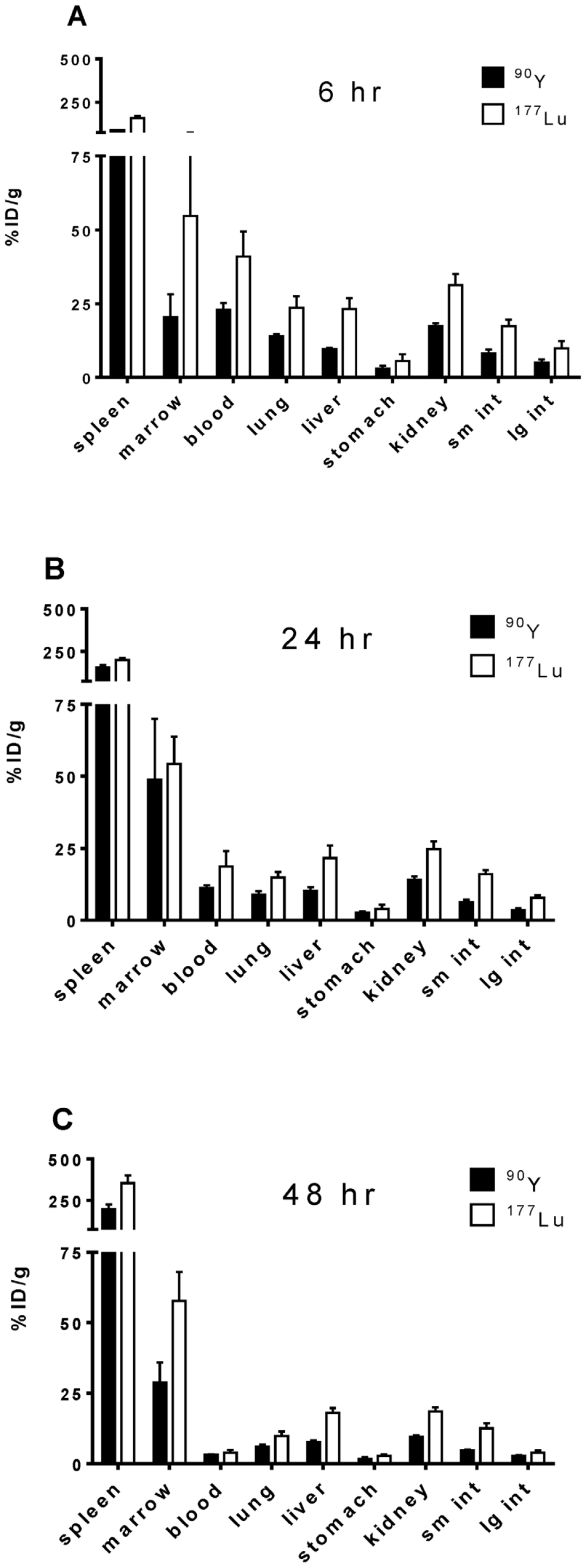
^177^Lu- and ^90^Y-DOTA-30F11 Biodistribution. Five mice per group received 10^5^ syngeneic leukemic cells *i.v.* two days before radiolabeled anti-CD45 DOTA-Ab. Mice were euthanized at **A**) 6, **B**) 24, and **C**) 48 hours after injection of (▪) ^90^Y- or (□) ^177^Lu- DOTA-30F11. Organs were harvested and counts measured to calculate % ID/g.

The biodistribution of ^90^Y- or ^177^Lu- radiolabeled anti-CD45 antibodies (DOTA-30F11) were compared in mice harboring syngeneic murine myeloid leukemia. B6SJLF1/J mice were given 10^5^ myeloid leukemia cells *via* tail vein injection and two days later ^90^Y- or ^177^Lu-DOTA-30F11 was administered. Mice were euthanized after 6, 24, or 48 hours and their organs were harvested and analyzed on a gamma counter to calculate the % ID/g. Biodistribution studies demonstrated excellent localization of both ^90^Y and ^177^Lu to the BM and spleen, with much less uptake in non-target (*i.e.*, non-hematolymphoid) organs. Although the majority of ^177^Lu-DOTA-30F11 Ab conjugate rapidly localized to spleen (158.7±12.2% ID/g) and BM (54.7±21.5% ID/g) after 6 hours, significant uptake of ^177^Lu-DOTA-30F11 was also noted in blood-rich organs such as lung (23.7±3.9% ID/g), liver (23.3±3.6% ID/g), and kidney (31.3±3.7% ID/g) at 6 hours. Less ^90^Y-DOTA-30F11 was present in non-target organs at 6 hours (lung: 13.9±0.8, p = 0.006; liver 9.6±0.44, p = 0.001; kidney 17.4±0.92% ID/g; p = 0.001 compared to ^177^Lu; Fig. 1A). The difference in organ uptake between ^90^Y and ^177^Lu was 9.8% ID/g (95%CI: [5.0–14.5], p = 0.0019) for the lung, 13.7% ID/g ([9.3–18.1], p = 0.0001) for the liver, and 13.9% ID/g ([9.3–18.5], p = 0.0002) for the kidneys. These differences between ^90^Y- and ^177^Lu-DOTA-30F11 localization persisted after 24 hours in non-hematopoietic organs, with 14.0±1.2% ID/g of ^90^Y-DOTA-30F11 compared to 24.8±2.6% ID/g of ^177^Lu-DOTA-30F11 (p<0.001) in the kidneys, with a difference of 10.8% ID/g ([7.8–13.7], p<0.0001). Differences in uptake between ^90^Y- and ^177^Lu-DOTA-30F11 delivered to BM and spleen were less pronounced after 24 hours (48.8±11 and 156±15% ID/g for ^90^Y-DOTA-30F11 and 54.2±9.6 and 199±11% ID/g for ^177^Lu-DOTA-30F11, in the BM and spleen, respectively; Fig. 1B). The uptake difference between ^90^Y and ^177^Lu at 24 hours was 5.4% ID/g ([21.9–32.8], p = 0.6522) in the BM and 43.3% ID/g ([24–62.7], p = 0.0009) in the spleen. Although the BM uptake appears to decrease for ^90^Y from 24 to 48 hours, the large errors bars suggest BM uptake was stable during this time. The highest uptakes of radiolabeled DOTA-Ab were observed in target tissues after 48 hours for both ^90^Y-DOTA-30F11 and ^177^Lu-DOTA-30F11 (Fig. 1C), although higher BM and spleen localization was seen for ^177^Lu-DOTA-30F11 (57.7±10 and 354±48% ID/g, respectively) compared to ^90^Y-DOTA-30F11 (28.8±7.1 and 197±29% ID/g, respectively). There was higher uptake of ^177^Lu compared to ^90^Y at the BM [difference of 28.9% ID/g ([16.0–41.8], p = 0.0008)] and spleen [difference of 157.6% ID/g ([100.1–215.1], p = 0.0002)]. These biodistribution data suggest that both radionuclides were effectively targeted to BM and spleen with minor uptake in non-hematologic organs.

**Figure 2:**
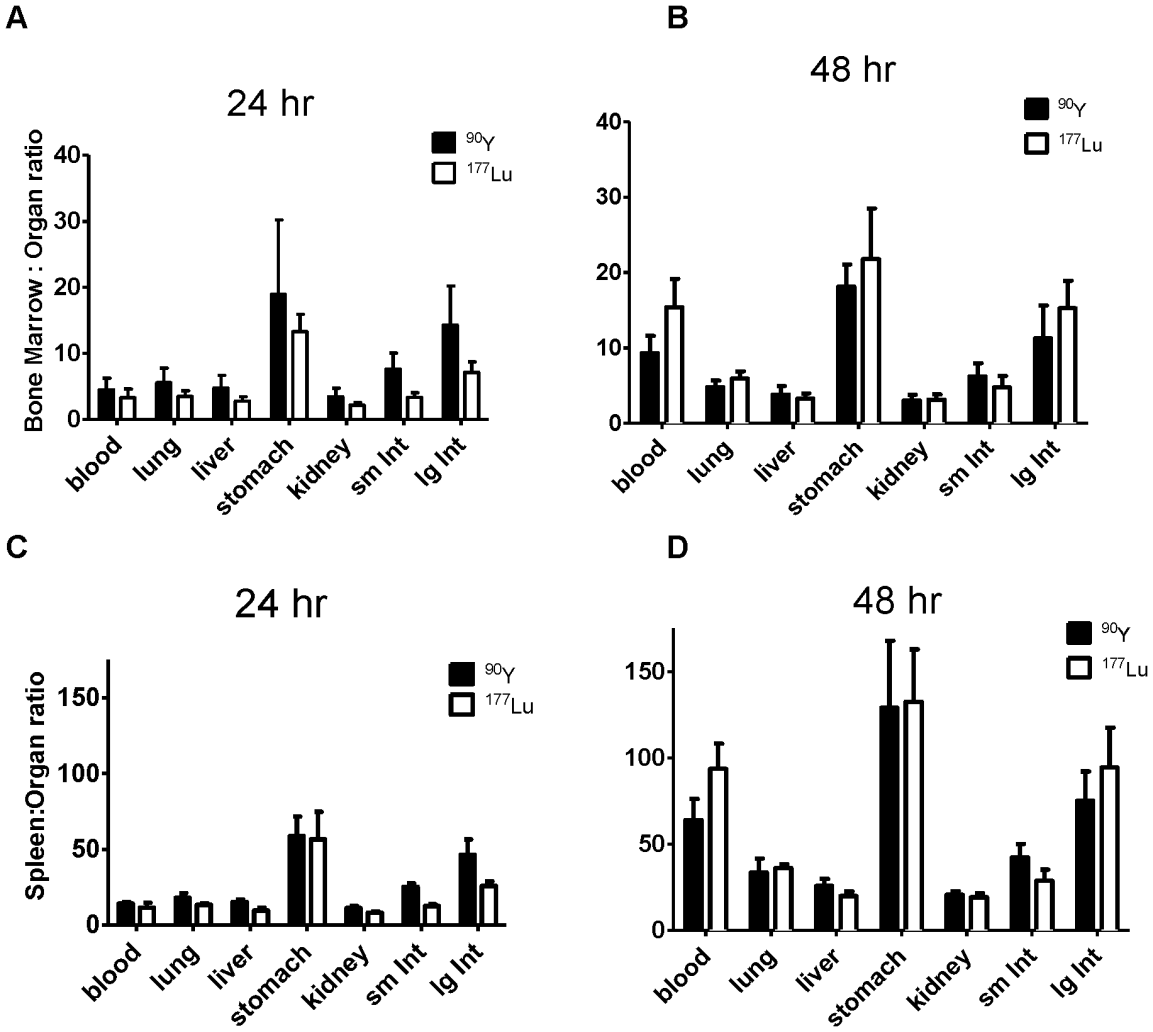
Target-to-Normal Organ Ratios. Mean ratios of radioactivity uptake for ^90^Y-DOTA-30F11 (▪) and ^177^Lu-DOTA-30F11 (□) delivered to BM (**A** and **B**) or spleen (**C** and **D**) compared to normal organs at 24 and 48 hours.

To underscore the preferential targeting of hematologic organs compared to normal organs, ratios of the % ID/g of target organs (BM and spleen) to normal organs were calculated for both ^177^Lu- and ^90^Y-radiolabeled Ab after 24 and 48 hours. Target-to-normal organ ratios for dose-limiting organs were higher but not statistically significant for ^90^Y-DOTA-30F11 than for ^177^Lu-DOTA-30F11 after 24 hr. with an average BM-to-lung ratio of 5.6∶1 compared to 3.5∶1 for ^177^Lu (p = 0.1747), and BM-to-liver ratios of 4.7∶1 and 2.8∶1 for ^90^Y and ^177^Lu (p = 0.0969), respectively (Fig. 2A). These ratios were stable or improved with time, presumably due to progressive blood clearance (Fig. 2B). Spleen-to-normal organ ratios were higher than BM-to-normal organ ratios for both ^90^Y and ^177^Lu (Fig. 2C and D), likely due to the higher density of CD45^+^ cells in the spleen in this leukemia model. Radiation-sensitive normal organs were relatively spared from excessive radiation exposure, with spleen-to-lung ratios of 18.0∶1 and 13.3∶1 after 24 hours for ^90^Y and ^177^Lu, respectively, and spleen-to-liver ratios of 15.3∶1 and 9.5∶1 for ^90^Y and ^177^Lu, respectively. Target-to-normal organ ratios improved 48 hours after radiolabeled Ab delivery, with spleen-to-lung ratios of 33.5∶1 and 36.1∶1 and spleen-to-liver ratios of 25.8∶1 and 19.9∶1 for ^90^Y-DOTA-30F11 and ^177^Lu-DOTA-30F11, respectively. In addition, the kidneys, a particular source of concern in radioimmunotherapy studies, showed favorable radiation exposure with BM-to-kidney ratios of 3.0∶1 and 3.1∶1 after 48 hours for ^90^Y and ^177^Lu, respectively, and spleen-to-kidney ratios of 20.6∶1 and 19.2∶1 for ^90^Y and ^177^Lu, respectively.

### Comparative *in vivo* Cerenkov Light Imaging of ^90^Y- and ^177^Lu-DOTA-30F11

**Figure 3:**
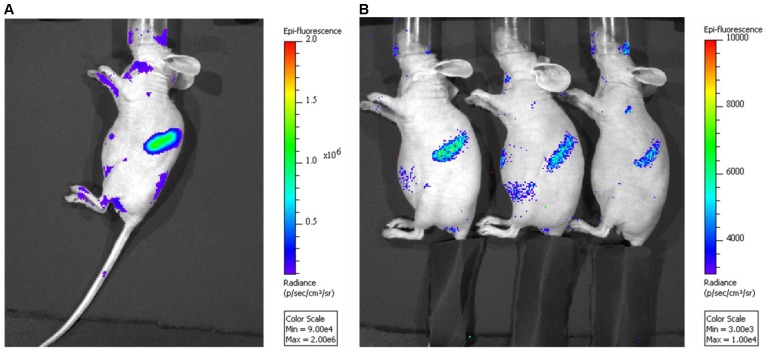
Cerenkov Imaging. Five female athymic mice per imaging group were injected with either **A**) 0.3 mCi of ^90^Y-DOTA-30F11 or **B**) 0.3 mCi ^177^Lu-DOTA-30F11 and imaged 24 hours after injection, using a Xenogen IVIS Spectrum imaging platform under 2.5% isoflurane. Differences in the intensity of splenic activity between the mice that received the two isotopes may be attributed to differences in the decay energies of their emitted β^−^ particles; note the different scales used in each image.

We sought to visualize the *in vivo* targeting of ^90^Y- and ^177^Lu-DOTA-30F11 *via* Cerenkov Light Imaging (CLI) experiments. Cerenkov radiation arises when charged particles such as β^−^, or β^+^ emissions travel through an optically transparent insulating material (typically water) with a velocity that exceeds the speed of light. As charged particles travel through water, they lose kinetic energy by polarizing the electrons of water molecules. Relaxation of the polarized molecules occurs *via* the emission of light energy, giving rise to the observed Cerenkov emission, or a continuous spectrum of light from near-ultraviolet to visible [26]–[29]. Athymic nude mice were injected with 300 µCi of either ^90^Y- or ^177^Lu-DOTA-30F11, and both radiolabeled Ab conjugates displayed distinct, focal localization to the spleen by CLI as early as 15 minutes post-injection. The spleens were the major organ of localization in all imaged mice, and the overwhelming splenic pixel intensity could have prevented visualization of other sites of uptake. As expected, imaging confirmed increased uptake in CD45^+^ organs, especially spleen and liver, for both radionuclides. However, ^90^Y-DOTA-30F11 treated mice had higher signals (peak radiance of 1.2×10^6^ p/sec/cm^2^/sr) than ^177^Lu- treated mice (peak radiance of 6.8×10^3^ p/sec/cm^2^/sr), likely because of the higher decay energy and longer path length of ^90^Y compared to ^177^Lu (Fig. 3).

### Radioimmunotherapy with ^90^Y-DOTA-30F11 versus ^177^Lu-DOTA-30F11

**Figure 4:**
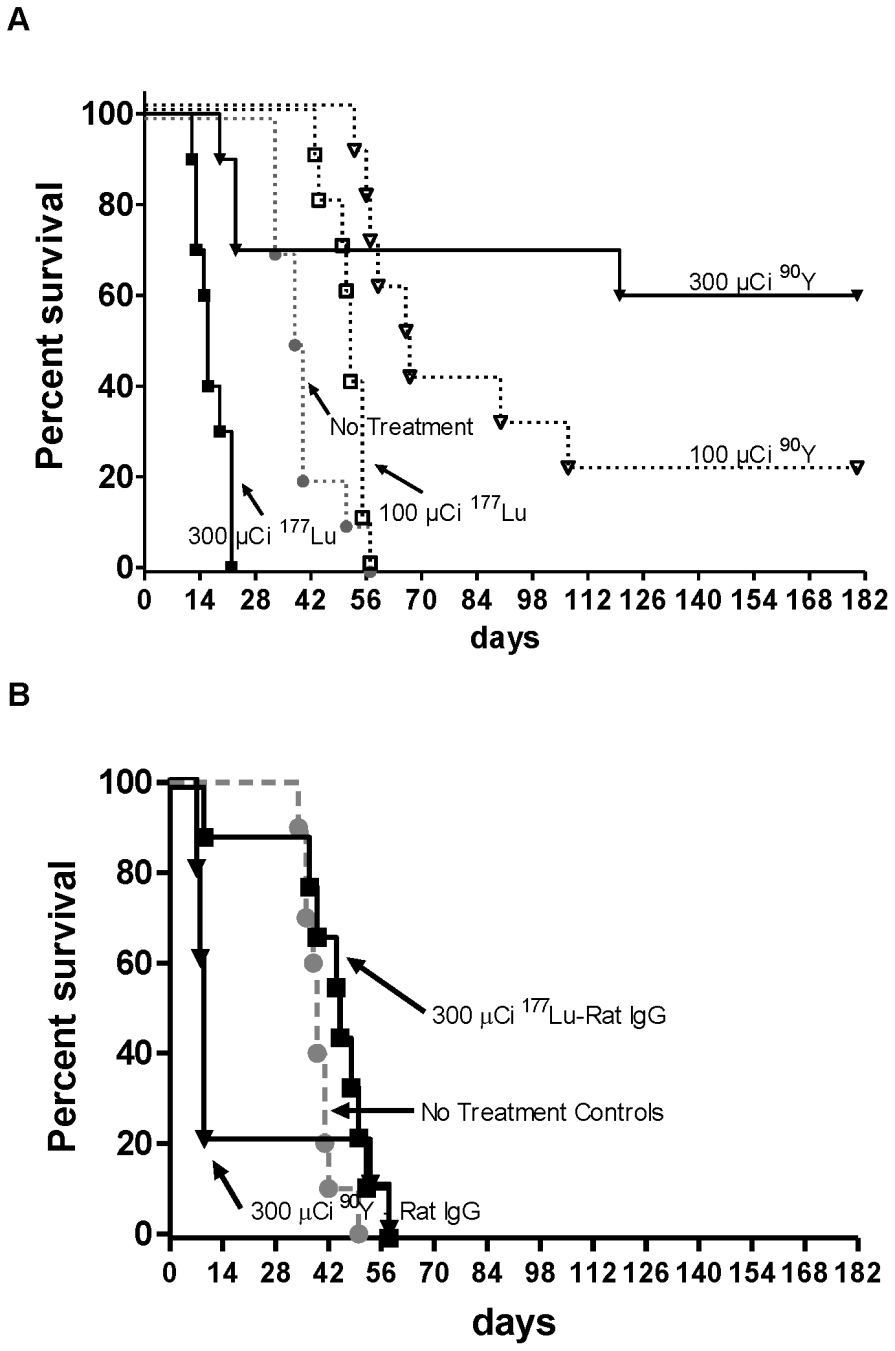
Survival from anti-CD45 RIT using ^90^Y or ^177^Lu. Ten mice per group were given 10^5^ syngeneic leukemic cells on day -2, and then injected with **A**) ^90^Y-(∇) or ^177^Lu-(□)-DOTA-30F11, at 300 µCi (filled), or 100 µCi (unfilled); or **B**) isotype control Ab ^90^Y-(▾)- or ^177^Lu-(▪) DOTA-rat IgG at 300 µCi, on day 0, and monitored for death, extensive weight loss or lethargy, in which case they were euthanized. Untreated leukemic mice (-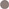-) served as a control group.

As both radionuclides effectively targeted CD45^+^ tissues *in vivo* we then tested ^90^Y- and ^177^Lu-DOTA-30F11 to treat disseminated syngeneic myeloid leukemia in mice. Ten mice per group were given 10^5^ SJL leukemic cells *via* tail vein injection and two days later were treated with 300 or 100 µCi of either ^90^Y- or ^177^Lu-DOTA-30F11. A prior pilot study demonstrated that 300 µCi of ^90^Y had previously been well tolerated by mice from a related SJL strain [19]. All ten untreated control mice died, with the median survival (the time at which the Kaplan-Meier estimate of survival reaches 50% or lower) being 39 days. Eight of the 10 mice that received 100 µCi ^90^Y-DOTA-30F11 died, with the median survival being 66 days, and 4 of the 10 mice that receive 300 µCi ^90^Y-DOTA-30F11 died with the median survival not reached (Fig 4A). These data showed a statistically significant trend in decreased mortality as dose increased (from control to 100 µCi to 300 µCi, p.003). On the other hand, all 10 mice that received 100 µCi ^177^Lu-DOTA-30F11 died as did all 10 mice that received 300 µCi ^177^Lu-DOTA-30F11 (median day of survival, 52 days, 16 days, respectively). There was actually a statistically significant trend for increased mortality as the dose of ^177^Lu-DOTA-30F11 increased. To confirm that the therapeutic benefits observed were due to targeting of radionuclides to the BM and spleen and not to a non-specific radiation effect, mice were also treated with an isotype negative control DOTA-rat IgG that was radiolabeled with 300 µCi of either ^90^Y or ^177^Lu (Fig. 4B). Therapy with 300 µCi ^90^Y-DOTA-rat IgG resulted in excessive toxicity with 80% (8/10) of mice requiring euthanasia for excessive weight loss, contributing to a median survival of 9 days after therapy. Mice treated with 300 µCi ^177^Lu-DOTA-rat IgG had a median survival of 45 days, with the deaths from progressive leukemia characterized by splenic enlargement from leukemic infiltration. These results suggest that ^90^Y-DOTA-30F11 improved survival in this leukemia model in a dose-dependent manner, while ^177^Lu-DOTA-30F11 was too toxic or ineffective at similar doses.

### Dosimetry Estimates of Absorbed Doses of Radiation Delivered using ^90^Y and ^177^Lu

The absence of a therapeutic benefit when using ^177^Lu-DOTA-30F11 despite similar targeting specificity of ^90^Y-DOTA-30F11 was unexpected. We hypothesized this might be due, at least in part, to differences between absorbed radiation doses in target organs such as the BM and spleen. Consequently, we estimated the absorbed radiation doses for organs of interest per unit of activity injected for both ^90^Y- and ^177^Lu-DOTA-30F11 [23]. Dosimetry calculations showed that the BM absorbed dose per µCi of ^90^Y-DOTA-30F11 injected was more than 11-times greater than the absorbed radiation dose delivered by ^177^Lu-DOTA-30F11 (8.5 *versus* 0.74 cGy/µCi; Table 1). Although not as dramatic, the spleen absorbed dose per unit injected was more than 2-fold higher for ^90^Y- than ^177^Lu-DOTA-30F11 (82.5 *versus* 31.9 cGy/µCi). All other organs from mice treated with ^90^Y-DOTA-30F11 had 2- to 4-fold increases in absorbed dose per unit injected compared to mice treated with ^177^Lu-DOTA-30F11. The markedly disparate absorbed doses between ^90^Y and ^177^Lu based anti-CD45 RIT could be due to the higher energy profile of ^90^Y (E_max_ = 2.3 MeV) compared to ^177^Lu (E_max_ = 0.5 MeV). The longer physical half-life of ^177^Lu (6.7 days *versus* 2.6 days for ^90^Y) did not compensate for the difference in energy profiles, contrary to our expectations. Lastly, the disparate absorbed doses may have contributed to the lack of efficacy at the lower dose (100 µCi) seen with ^177^Lu-DOTA-30F11 in this model, while the higher dose tested (300 µCi) was excessively toxic to the BM, possibly from the longer half-life of ^177^Lu interfering with hematopoiesis.

**Table 1:** Calculated absorbed dose (cGy)/µCi administered.

Organ	^90^Y	^177^Lu
**Bone Marrow**	8.54	0.743
**Spleen**	82.5	31.92
**Kidney**	10.9	2.78
**Liver**	5.12	2.31
**Lungs**	5.23	1.92
**Stomach**	4.75	2.32
**Sm. Intestine**	5.55	1.7
**Lr. Intestine**	3.37	0.918
**Blood**	2.9	2.27
**Tail**	2.85	0.687
**Muscle**	1.85	0.166

### Assessment of Toxicity after RIT using ^90^Y- versus ^177^Lu-DOTA-30F11

**Figure 5:**
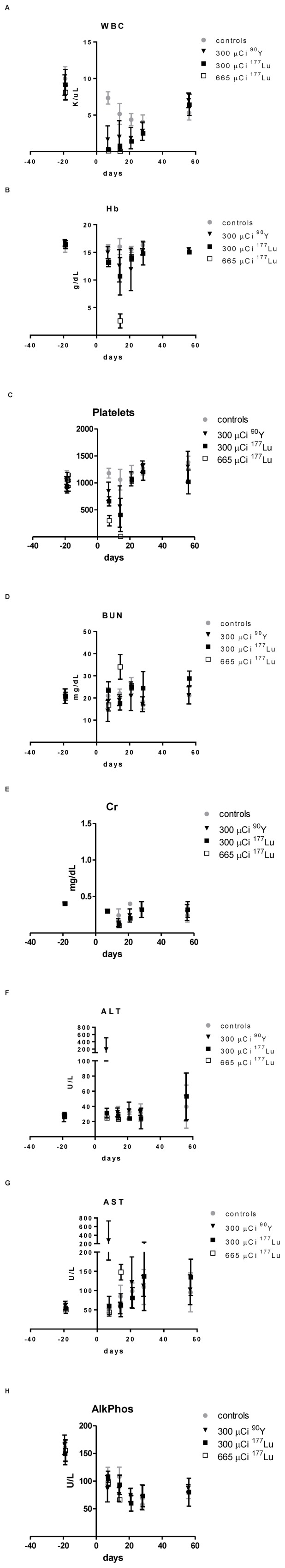
Hematologic, renal and hepatic toxicity. Five mice per group were bled at baseline (day -19), 1, 2, 3, 4, and 8 weeks after injection of radiolabeled anti-CD45 Ab at time 0 [300 µCi ^90^Y-DOTA-30F11 (▾), 300 µCi ^177^Lu-DOTA-30F11 (▪), or liver ^90^Y-DOTA-30F11 equivalent dose of 665 µCi ^177^Lu-DOTA-30F11 (□)]. Blood was analyzed for **A**) WBC, **B**) Hg, **C**) platelets, **D**) BUN concentrations, **E**) Cr, **F**) AST, **G**) ALT, and **H**) ALP, and values were compared to values from age-matched untreated control mice (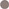).

To evaluate the tolerability of ^90^Y- and ^177^Lu-anti-CD45 RIT, mice were given 300 µCi ^90^Y- or ^177^Lu- DOTA-30F11. Because these injected doses do not yield comparable absorbed radiation doses in dose-limiting organs, an equivalent liver absorbed dose was also used in toxicity studies (665 µCi ^177^Lu-DOTA-30F11, the equivalent liver absorbed dose to 300 µCi ^90^Y-DOTA-30F11). Laboratory studies were performed before the start of therapy, 1, 2, 3, 4 and 8 weeks after injection of each radiolabeled DOTA-30F11 conjugate. Blood was analyzed for complete blood counts and renal and hepatic functions. The most pronounced toxicity detected with both radionuclides was myelosuppression with minimal impact on renal or hepatic functions. The baseline white blood cell count (WBC) in untreated control animals was 7.3±0.8 K/µL. Mice treated with anti-CD45 RIT exhibited an expected leucopenia 1 week after injection of radiolabeled DOTA-30F11 with a WBC nadir of 1.7±1.9 (23.3% of untreated controls), 0.3±0.1 (4.1% of untreated controls), and 0.1±0.03 K/µL (1.4% of untreated controls) for mice treated with 300 µCi ^90^Y-, 300 µCi ^177^Lu-, and 665 µCi ^177^Lu-DOTA-30F11, respectively (p = 0.5046; Fig. 5A). Mice treated with 665 µCi ^177^Lu-DOTA-30F11 developed fatal anemia and thrombocytopenia as these mice died by week 2 after RIT injections [mean hemoglobin (Hb) 2.6±1.3 g/dL (16.3% of untreated controls), mean platelet count 7.3±1.8 K/µL (0.7% of untreated controls), compared to mean Hb 16.0±1.5 g/dL [difference of 13.4 g/dL ([11.3-15.6], p<0.0001)] and mean platelets 1061.2±191 K/µL in untreated control mice [difference of 1054.0 K/µL ([824.5–1283.4], p<0.0001)]; Fig. 5B and C]. Mice treated with 665 µCi ^177^Lu-DOTA-30F11 had evidence of gastrointestinal bleeding on gross necropsy, as no histological analysis was done on tissues. However, mice treated with 300 µCi of either ^90^Y- or ^177^Lu-DOTA-30F11 showed mild transient anemia [mean Hb 12.6±2.9 g/dL (78.8% of matched controls) and 10.7±3.4 g/dL (66.9% of matched controls), [difference of 1.9 g/dL ([2.7–6.5], p = 0.3747)], respectively, and mild thrombocytopenia with mean platelets of 562.8±383 K/µL (53.0% of matched controls) and 406.4±308.0 K/µL (38.3% of matched controls) [difference of 156.4 K/µL ([350.5–663.3], p = 0.4970)], respectively]. By week 4, the complete blood counts from mice treated with 300 µCi of ^90^Y- and ^177^Lu- 30F11-DOTA were similar to blood counts from untreated mice (p = 0.08 by one way ANOVA), and remained within normal range through week 8.

Additional experiments showed minimal renal or hepatic toxicity from ^90^Y- and ^177^Lu-anti-CD45 RIT in this model. Serum Cr and BUN values remained within normal limits throughout the study (<0.5 mg/dL and 10–36 mg/dL, respectively; Fig. 5D and E). Mice treated with 665 µCi of ^177^Lu-DOTA-30F11 had higher BUN levels (mean 34±5.6 mg/dL) at week 2, with a difference of 12 mg/dL ([5.6–18.4], p = 0.0039) compared to untreated control mice, likely from gastrointestinal bleeding. Minimal hepatic dysfunction was detected after RIT with either radionuclide, although mice treated with 665 µCi of ^177^Lu-DOTA-30F11 exhibited slightly increased AST levels at week 2 after therapy (mean 147.7±20.2 U/L), compared to the mice treated with 300 µCi of ^90^Y [mean AST 55.0±14.9; for a difference of 85.9 U/L ([37.5–134.2], p = 0.0049), Fig. 5G]. Other liver function tests, such as ALT (Fig. 5F), and ALP (Fig. 5H) from RIT treated mice did not differ from untreated matched control mice throughout the 8 weeks of monitoring. In summary, toxicity studies showed transient myelotoxicity with anti-CD45 RIT that normalized by 4 weeks after therapy, with minimal hepatic or renal toxicity.

## Discussion

The results suggest that ^90^Y-anti-CD45 RIT was more effective than ^177^Lu-anti-CD45 RIT to treat leukemia in a syngeneic murine model (Fig. 4). Biodistribution studies showed both ^90^Y- and ^177^Lu-DOTA-30F11 localized similarly to target sites with the highest disease burden, BM and spleen (Fig. 1). *In vivo* Cerenkov imaging (Fig. 3) allowed visualization of radiolabeled DOTA-30F11 with both β^-^ emitting radionuclides localizing to the spleens, confirming specific targeting by biodistribution studies. Furthermore, anti-CD45 RIT using 300 µCi ^90^Y or ^177^Lu was well tolerated, since the most pronounced toxicity was transient myelosuppression without renal or hepatic dysfunction (Fig. 5). Thus, the lack of efficacy from ^177^Lu-DOTA-30F11 was not from an inability to deliver ^177^Lu to sites of disease, but may be explained by their differences in physical energy properties. Residence time of ^90^Y- or ^177^Lu-DOTA-30F11 should not have varied, as residence time is more dependent on the stability of the bioconjugate than on the specific radionuclide. Treatment with ^177^Lu should have concentrated the decay energies closer to target tissues than ^90^Y given the shorter path length of ^177^Lu (0.9 mm) compared to that of ^90^Y (2.7 mm), according to theoretical calculations. If we consider absorption energies in hypothetical spheres of 0.1, 1 and 10 mm diameter, because of the effective path length differences, the fraction of energy deposited in these spheres would be 1, 9, and 66% for ^90^Y compared to 15, 67, and 97% for ^177^Lu, respectively [30], [31]. Unfortunately, their decay energies are significantly different such that even if this schema was followed the absolute energy differences may not have allowed for comparable radiation doses. ^177^Lu has a low energy gamma-radiation fraction (0.2 MeV) with the majority in the beta range (78.6% at 0.5 MeV) and a mean energy beta-radiation of E_mean_ = 0.13 MeV [32]. For comparison, the mean energy from ^131^I beta-radiation is about E_mean_ = 0.18 MeV while the E_mean_ of ^90^Y = 0.9 MeV [31]. Taken together, the mean beta-particle energy per decay emitted by ^90^Y appears to be 7-fold higher than that of ^177^Lu, resulting in lower effective radiation doses to target tissues. Dosimetry calculations support this hypothesis as absorbed doses in tissues from mice treated with ^90^Y-DOTA-30F11 were 2- to 11- fold higher than mice treated with ^177^Lu-DOTA-30F11 (Table 1). The efficacy differences between ^90^Y- and ^177^Lu-DOTA-30F11 may be explained, at least partially, by the large differences in decay energy that lead to disparate absorbed doses of the two radionuclides.

Absorbed doses were lower in ^177^Lu-DOTA-30F11 treated mice compared to mice treated with ^90^Y-DOTA-30F11 when each group received 300 µCi of radioactivity. However, mice given an equivalent liver absorbed dose (665 µCi of ^177^Lu-DOTA-30F11) had fatal hematologic toxicities, suggesting that absorbed dose alone may not explain the differential efficacy. Mice treated with 665 µCi ^177^Lu-DOTA-30F11 received 212 Gy of radiation delivered to the spleen compared to a similar dose of 248 Gy delivered to the spleen when treated with 300 µCi ^90^Y-DOTA-30F11. Consequently, a correlate explanation for the efficacies may lie in dose rate differences; at the spleen, 248 Gy from 300 µCi of ^90^Y-DOTA-30F11 was delivered at a higher dose-rate over a smaller time frame given the shorter half-life of ^90^Y, compared to the 212 Gy from 665 µCi ^177^Lu-DOTA-30F11 at a lower dose-rate over its longer half-life. Indeed, clinical trials have shown increased relapse rates with increased dose fractionation and reduced dose-rates [33]–[35]. Similarly, murine transplant models that increased dose fractionation or lowered dose-rate of radiation effectively restored host hematopoiesis, and required higher total TBI doses for donor engraftment [36], suggesting that lower dose-rates may significantly lower the cytotoxic effect of radiation.

Lastly, these results also assessed the potential long-term toxic effects of ^90^Y- *versus* ^177^Lu-anti-CD45 RIT. Mice without leukemia used in toxicity studies may explain why mice given 300 µCi ^177^Lu-DOTA-30F11 did not display the fatal toxicities that were seen in the RIT studies employing disease-bearing animals. While speculative, leukemic cells used for RIT experiments provided additional CD45^+^ target, which lead to more radioactivity in hematolymphoid tissues producing more hematologic toxicity. Further, myelotoxicity could have been more pronounced with ^177^Lu because the longer half-life interfered with effective hematopoiesis, risking potentially fatal infections, lethal anemia, and/or bleeding complications.

In summary, these studies in a syngeneic disseminated leukemia model confirm the therapeutic efficacy of ^90^Y-anti-CD45 RIT for leukemia but do not support the addition of ^177^Lu to RIT treatment options. The lack of efficacy using ^177^Lu was not due to suboptimal targeting as both radionuclides were delivered equally to BM and spleen, but may have been due to differences in radiation properties, such as decay energies, effective path-lengths, and dose-rates. The longer half-life of ^177^Lu was unable to provide a comparable absorbed dose of ^90^Y, and in fact its longer half-life and radiation effects may have interfered with effective hematopoiesis and further added to its myelotoxicity.
